# Towards a Consensus on an ICF-Based Classification System for Horizontal Sound-Source Localization

**DOI:** 10.3390/jpm12121971

**Published:** 2022-11-29

**Authors:** Griet Mertens, Ellen Andries, Anja Kurz, Dayse Tȧvora-Vieira, Miryam Calvino, Edda Amann, Ilona Anderson, Artur Lorens

**Affiliations:** 1 Department of Otorhinolaryngology, Head and Neck Surgery, Antwerp University Hospital, 2000 Antwerp, Belgium; 2 Experimental Laboratory of Translational Neurosciences and Dento-Otolaryngology, Faculty of Medicine and Health Sciences, University of Antwerp, 2000 Antwerp, Belgium; 3 Department of Otorhinolaryngology, Plastic, Aesthetic and Reconstructive Head and Neck Surgery, Comprehensive Hearing Center, University Hospital Würzburg, 97078 Würzburg, Germany; 4 Otolaryngology, Head & Neck Surgery, Medical School, Division of Surgery, The University of Western Australia, Perth 6150, Australia; 5 Fiona Stanley Fremantle Hospitals Group, Perth 6150, Australia; 6 School of Occupational Therapy, Social Work and Speech Pathology, Faculty of Health Sciences, Curtin University, Perth 6150, Australia; 7 Department of Otolaryngology, Hospital Universitario La Paz, 28046 Madrid, Spain; 8 Biomedical Research Networking Centre on Rare Diseases (CIBERER), Institute of Health Carlos III, 28029 Madrid, Spain; 9 Department of Clinical Research, MED-EL Medical Electronics, 6020 Innsbruck, Austria; 10 World Hearing Center, Institute of Physiology and Pathology of Hearing, 05-830 Kajetany, Poland

**Keywords:** sound localization, binaural hearing, cochlear implant, interaural level difference, interaural time difference, single-sided deafness, unilateral hearing loss, testing-method consensus, classification, ICF

## Abstract

The study aimed to develop a consensus classification system for the reporting of sound localization testing results, especially in the field of cochlear implantation. Against the background of an overview of the wide variations present in localization testing procedures and reporting metrics, a novel classification system was proposed to report localization errors according to the widely accepted International Classification of Functioning, Disability and Health (ICF) framework. The obtained HEARRING_LOC_ICF scale includes the ICF graded scale: 0 (no impairment), 1 (mild impairment), 2 (moderate impairment), 3 (severe impairment), and 4 (complete impairment). Improvement of comparability of localization results across institutes, localization testing setups, and listeners was demonstrated by applying the classification system retrospectively to data obtained from cohorts of normal-hearing and cochlear implant listeners at our institutes. The application of our classification system will help to facilitate multi-center studies, as well as allowing better meta-analyses of data, resulting in improved evidence-based practice in the field.

## 1. Introduction

### 1.1. The Importance of Sound Localization

Sound localization is an important skill for perceiving and navigating the world around us. Fundamentally, localization allows one to determine the direction and distance of a sound source. This facilitates auditory scene analysis and selective attention [1,2], as well as specific skills such as demasking of speech in noise [3] and perception of auditory motion [4].

### 1.2. Physiological Mechanism of Sound Localization

Distinct physiological mechanisms underlie sound localization in the horizontal (azimuthal) and vertical domains. Humans live in a largely planar world and are considerably more adept at localizing sounds in the horizontal domain than in the vertical domain [5]. Consequently, most efforts in the restoration of binaural cues focus on measuring and improving horizontal localization. The two main binaural cues for horizontal localization are interaural level differences (ILD) and interaural time differences (ITD). ILDs are produced by the head shadow effect, which occludes higher frequencies with wavelengths shorter than head diameter (>1500 Hz). At these frequencies, ILDs dominate horizontal localization processing [6]. Along with monaural spectral, level, and reverberation cues, ILDs also contribute to judging the distance to a sound source [5]. ITDs are produced as a consequence of the difference in arrival time at each ear when the source is not located at the interaural midline. For periodic stimuli such as pure tones, this is equivalent to an interaural phase difference (IPD). ITDs are the dominant cue for low frequency sounds (<1500 Hz). Most natural sounds contain both high- and low-frequency components, so ITDs and ILDs must be integrated to form a unified neural representation of the position of a sound source in the horizontal domain. The main locus for this processing is the superior olivary complex [6]. Localization in the vertical domain is dominated by the monaural spectral deformation cues provided by head-related transfer functions, which are unique to each individual [5]. As this paper focuses on horizontal localization, these will not be discussed further here.

### 1.3. Factors Which Affect the Accuracy of Sound Localization

The accuracy of localization depends on several factors. First is the azimuthal position of the sound source: sounds presented at the interaural midline are more accurately localized than those presented laterally, and frontally presented stimuli are somewhat more accurately localized than those presented from behind [5]. The characteristics of the acoustic stimulus are another important factor. Localization is most accurate for low-frequency sounds (<1000 Hz) and least accurate for stimuli with frequencies between 1000 and 3000 Hz. Spectral width also has an effect, with wider-band sources being more accurately localized. Better performance is seen for speech sounds than for tones. Head movements also provide monaural and binaural dynamic cues. Sound level, duration, and envelope modulation have little influence upon localization [5].

### 1.4. Sound-Localization Ability and Hearing Loss

Sound-localization ability is often compromised by hearing loss. Asymmetric or bilateral hearing loss disrupts the delivery of binaural cues to higher neural structures for integration and processing. Peripheral hearing loss for an extended duration can also induce plastic changes in central hearing pathways, further compounding the issue [7]. One of the major goals of hearing-assistive technologies is to restore sound-localization ability in people with hearing loss. There is substantial clinical evidence that hearing-assistive technologies support binaural hearing, resulting in improved sound localization [8]. Bilateral cochlear implant (CI) users can achieve performance within the range of individuals with normal hearing (NH) [8,9]. Bimodal users with a contralateral hearing aid also benefit, albeit to a lesser degree. These results are at odds with earlier thinking in the field; previously, it was believed that bimodal users were disadvantaged for binaural hearing. It was though that integrating the input from two differently impaired and differently rehabilitated ears was too complex of a challenge for the brain [6]. In individuals with unilateral hearing loss or single-sided deafness (UHL/SSD), unilateral CI use has been shown to restore binaurality and sound localization [10]. The number of cochlear implantations performed annually has steadily increased [11], driven in part by increased awareness in the community, reduced costs, and technological, surgical, and rehabilitative improvements that yield better hearing outcomes. With the expansion of candidacy criteria to younger children, individuals with SSD or UHL, and those with a greater degree of residual hearing, this trend is set to further increase in the future. Although significant and meaningful benefits to binaural hearing restoration are achieved with CI use, there is a large degree of variation in localization outcomes. This is believed to depend on device hardware and engineering, pathology of the auditory system, speech processing strategies used, microphone characteristics, and other factors [6,8,10]. Much research is still needed to bring these outcomes closer to normal performance and to monitor the trajectory of hearing rehabilitation of current CI users.

### 1.5. Measures of Sound Localization

The ability of an individual to localize a sound source can be assessed using subjective or objective measures. Subjective measures employ tools such as questionnaires and other self-reported metrics. One such commonly used tool is the Speech, Spatial and Qualities of Hearing Scale (SSQ) [12]. In these measures, individuals report their own performance in distinct scenarios, such as identifying whether a vehicle is approaching or receding, or judging the distance of footsteps or voices. Subjective measures are able to capture performance in scenarios that are difficult to replicate in a formal testing environment. Objective measures use psychophysical testing procedures to assess localization accuracy. These procedures take place in a formal testing environment and yield direct quantitative measures of sound localization. In the most common experimental paradigm, the listener is seated before an array of loudspeakers arranged in a semicircle. A sound is presented from one loudspeaker, and the listener is asked to identify which loudspeaker is the perceived source. The difference in azimuthal angle between the presenting loudspeaker and perceived loudspeaker can be quantified as the horizontal localization error. The smaller the error in degrees, the better the localization accuracy. 

### 1.6. Variations in Localization Test Procedures

The procedural elements of localization testing vary across institutions, and this can reduce the ability to directly compare results. Some examples of variations among recently published studies are outlined in Table 1 [10,13,14,15,16,17,18,19,20,21,22,23,24,25,26]. Sources of procedural variation include (but are not limited to):

(a) The geometry of the testing environment, such as the physical dimensions of the space, the use of anechoic rooms versus testing booths, etc.
(b) The type, number, and location of sound sources, the distance of the loudspeakers to the listener, the inter-speaker angle, etc.
(c) The type, number, presentation level, and duration of sound stimuli, which can be pure tones, wide- or narrow-band noise, speech-shaped noise, babble, individual human voices or conversations, or environmental sounds.
(d) The manner of listener response, such as verbal identification of the source speaker according to a pre-arranged naming scheme (*1-2-3*, *A-B-C*, etc.) or according to the analog clock scheme, or by pointing with hands or other instruments, or the use of a point-and-click computer interface or drawings on paper.

**Table 1:** Examples of variations in localization testing procedures and reporting metrics from recent studies. NR: not reported, LP: low-pass, HP; high-pass, BB, broadband noise.

Publication	Response Points	Stimulus Type	Stimulus Duration (s)	Stimulus Level (dB SPL Unless Otherwise Stated)	Presentations per Speaker	Number of Listeners	Listener Type (s)	Speaker Azimuth Range	Number of Speakers	Distance the Listener to Speaker (m)	Reporting Metric
Ausili et al., 2020 [13]	NR	LP, HP, and BB	0.2	50–70 dBA	NR	25	BiCI	±75°	5	1.5	MAE
Brown, 2018 [14]	NR	Gaussian	0.5	62–68	NR	6	BiCI	±90°	13	1.7	RMSE
Ehrmann-Mueller et al., 2020 [15]	NR	CCITT	NR	55–70	NR	7	UHL/SSD	±90°	3–9	NR	RMSE
Grantham et al., 2012 [16]	54	Human speech	0.3; 1.25	60	9	12	UHL/SSD	±84°	9	1.9	RMSE
Grossmann et al., 2016 [17]	135	CCITT	1.0	55–65	15	12	UHL/SSD	±90°	9	1.5	RMSE
Killan et al., 2019 [18]	30	Human speech	NR	70 dBA	6	127	BiCI	±60°	5	1.5	RMSE
Kurz et al., 2020 [19]	NR	CCITT	1.0	65–75 dBA	6	23	UHL/SSD	±90°	9	1.5	RMSE
Kurz et al., 2021 [20]	54	CCITT	1.0	65–75 dBA	6	29	UHL/SSD	±90°	9	NR	RMSE
Lopez-Poveda et al., 2019 [21]	88	BB	0.2	68–72	8	12	BiCI	±75°	Virtual	1.0	RMSE
Lorens et al., 2021 [22]	242	Envr. sounds	NR	NR	22	11	UHL/SSD	±50°	11	1.0	RMSE
Marx et al., 2021 [23]	NR	White	0.4	NR	NR	51	SSD and AHL	±90°	7	0.6	RMSE
Mertens et al., 2016 [10]	27	LP, HP, and BB	0.2	54–66	9	10	UHL/SSD	±90°	9	0.8	RMSE
Mertens et al., 2017 [24]	54	CCITT	1.3	70–80	6	23	SSD and AHL	±90°	9	NR	MAE
Mertens et al., 2018 [25]	7	CCITT	1.0	70–80	6		UHL/SSD	±90°	7	0.8	MAE
Nopp et al., 2004 [26]	135	CCITT	1.0	60–80	15	20	BiCI	±90°	9	NR	RMSE
Seebacher et al., 2019	84	CCITT	1.3	65–75 dBA	12	12	UHL/SSD	±90°	7	1.0	RMSE
Seebacher et al., 2022	84	LP, HP, and BB	1.3	65–75 dBA	12	12	UHL/SSD	±90°	7	NR	RMSE
Skarzynski et al., 2017 [27]	141	Envr. sounds	NR	NR	2		UHL/SSD	±50°	11	2.0	RMSE
Tȧvora-Vieira et al., 2015 [28]	NR	4 kHz sound	NR	NR	NR	29	UHL/SSD	±60°	Virtual	NR	RMSE
Thompson et al., 2022 [9]	132	BB	0.2	52–72	12	20	UHL/SSD	±90°	11	1.0	RMSE
van Hoesel et al., 2003	40	Pink	0.8	61–69	5	20	BICI	±54°	8	1.4	RMSE
Wedekind et al., 2020	NR	4 kHz sound	NR	NR	NR	29	UHL/SSD	±60°	Virtual	NR	RMSE
Speck et al., 2021	NR	Human speech	NR	59–71	NR	51	SSD and AHL	±90°	7	NR	MAE
Dorbeau et al., 2018	≥48	Gunshot	NR	59–71	12	18	UHL/SSD	±90°	12	1.0	RMSE
Yost et al., 2013	132	LP, HP, and BB	0.2	65	12		NH	±77°	11	1.6	RMSE

Not all aspects of localization testing can or should be standardized across institutions. For example, the geometry of the testing environment is usually fixed. Similarly, different stimulus types may be appropriate for different clinical or research questions. Nevertheless, some procedural aspects can be standardized, and recommended procedures have been published elsewhere [29].

### 1.7. Variations in Localization Test Reporting Metrics

Numerous metrics have been used to calculate and report localization error in clinical studies and psychophysical research literature. The use of different metrics hinders the comparability of results across institutions. These metrics include the mean error (ME), standard deviation (SD), mean absolute deviation (MAD), mean absolute error (MAE), and root-mean-squared error (RMSE). These metrics are not necessarily compatible, and measure subtly different properties of localization ability. ME measures systematic error, which is the degree to which the perceived sound location differs to the actual sound location. It is roughly equivalent to accuracy and can be influenced by asymmetries in the listener’s hearing physiology or hearing technologies, leading to a non-centered auditory image. SD and MAD are measures of precision, which is the dispersion of perceived sound locations relative to the actual sound location. These metrics are influenced by factors such as the overall function of the listener’s hearing, as well as listener uncertainty and fluctuations in attention. MAE and RMSE are compound metrics which incorporate both systematic error and dispersion. In both metrics, listener performance is compared to a theoretical model (perfect performance, where the perceived loudspeaker is always the same as the presentation speaker). It has been argued that MAE is superior to RMSE as a model-performance measure [30]. Since RMSE relies on the sum of squared errors, it gives a higher weight to larger errors, meaning that extreme outlier responses can distort the overall measure to a greater degree. In localization testing, such extreme outliers occur in the form of reversal errors, where the listener perceives a sound source as coming from the opposite direction to its true location. These can occur in reverberant testing environments [31], or with the use of CIs, which give more limited spectral information and deprive the user of pinna cues [32]. Compared to MAE, RMSE also increases to a larger degree with increasing sample size, making comparisons difficult between studies with different numbers of participants or presentation–response trials.

### 1.8. A Classification System for Quantifying Localization Error Based on the ICF Framework

In this paper, we propose a novel classification system for reporting localization accuracy from experimental testing procedures. This will be used as the basis for the development of a universal scale to allow comparison of results from different institutions. The World Health Organization (WHO) International Classification of Functioning, Disability and Health (ICF) provides a framework for qualifying functioning and disability as well as the effectiveness of therapeutic interventions. Therefore, the use of the ICF framework provides a standard language to report on localization accuracy from different experimental testing procedures. A graded scale is used: 0 (no impairment), 1 (mild impairment), 2 (moderate impairment), 3 (severe impairment), and 4 (complete impairment). Core sets are provided for the classification of hearing loss [33], including *Localization of sound source (b2302)*. The final consensus classification system, here referred to as the HEARRING_LOC_ICF scale, will improve the comparability of localization test results across institutes, localization testing setups, and listeners. 

## 2. Methods

### 2.1. Development of the Consensus 

The HEARRING group is a network of world leading centers and experts dealing with all aspects of hearing disorders. We believe that advancements in the field of hearing devices are achieved through international research and the pooling of collective experience. At our annual meeting in November 2017 in Perth, Australia, we concluded that currently there exists no consensus on which outcome measures and methods are the most appropriate to use during CI evaluation and follow-up. Most CI outcome measures also require specific background knowledge in their interpretation, which complicates multidisciplinary collaboration and communication in the multidisciplinary rehabilitation process. At the same meeting, the International Classification of Functioning, Disability and Health (ICF) model was discussed as a valuable tool to overcome these challenges. To study the possibilities of the ICF framework within the field of CI, an ICF core team was put together. In the subsequent meetings of the ICF core group in Innsbruck (in 2018 and 2019), Vienna (in 2018), and Antwerp (in 2020 and 2022), ICF core sets were discussed for severe to profound sensorineural hearing loss and cochlear implantation, since they were non-existent at that time. The primary aim of the ICF core group was to define an ICF core set for CI users, based on the ICF core set for hearing loss, including all relevant ICF categories to describe the impact of cochlear implantation on different aspects of health. In a first phase, an overview of which domains of functioning that should be measured was collected. Subsequently, valid assessment tools for each ICF category were discussed, and we aimed to align all methods across the different centers cooperating in this study. The quantifications for the selected questionnaires, pure tone audiometry, and speech audiometry were developed based on the ICF qualifier categorization suggested by the WHO and on the clinical experience of the involved experts in this study. As different localization setups are used in the clinics for objective localization testing, the ICF qualifier quantification was calculated separately per particular localization setup based on norm level and chance level and will be discussed in this paper. In a third ongoing phase, we aim to apply the newly developed ICF-based assessment protocol, including the HEARRING_LOC_ICF scale in clinical practice by using it to assess adult CI users before and 6 months after implantation in all participating centers. The results of this prospective longitudinal multicenter study in 63 adult CI candidates will be presented in an upcoming paper.

### 2.2. Calculation of the HEARRING_LOC_ICF Scale 

**Figure 1:**
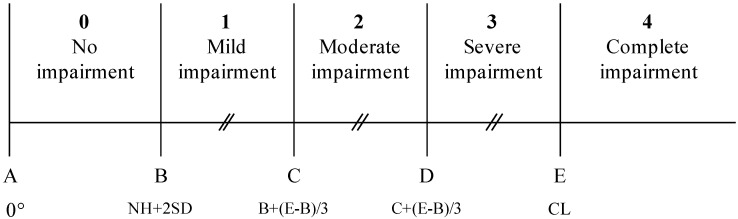
Schematic of the levels of impairment according to the ICF classification of localization ability. NH: Normal Hearing, CL: Chance level of the setup.

The proposed HEARRING_LOC_ICF scale uses the five-point graded scale from the ICF model to indicate the presence and degree of impairment in sound source localization. The categories are bounded by the norm data (localization error) of normal hearing population of each specific setup (NH) and chance level (CL) of the setup. The chance level of the localization error is calculated as the mean of the magnitudes of the differences between the azimuth of a judged speaker and the azimuth of a presenting speaker across all possible combinations of presenting and judged speakers. Consequently, chance levels and norm values will vary based on the localization testing setup, so these values will need to be calculated for each experimental setup separately. Formally, the five categories can be described as: **No impairment:** Performance ranging from 0° (perfect localization) to two standard deviations above the mean localization error of the normal-hearing group (NH + 2SD); **Mild impairment:** Performance ranging from NH + 2SD to NH + 2SD + ((CL − NH + 2SD)/3); **Moderate impairment:** Performance ranging from NH + 2SD + ((CL − NH + 2SD)/3) to NH + 2SD + (2 × (CL − NH + 2SD)/3); **Severe impairment:** Performance ranging from to NH + 2SD + (2 × (CL − NH + 2SD)/3) to chance level (CL); and **Complete impairment:** Performance at or worse than chance level (schematically outlined in Figure 1). Data should be produced with the same procedural setup using both a population of NH persons and the population of hearing-impaired persons from whichever population is of interest, such as unilateral or bilateral CI users. Since different metrics (for example, MAE and RMSE) measure subtly different properties, it is important to use norm data that were retrieved in the exact same setting, using the exact same metrics to calculate the HEARRING_LOC_ICF scale for that specific setup.

### 2.3. Institutes, Localization Testing Setups, and Listeners

In order to demonstrate the improvement of comparability of localization results across individuals, populations, and institutes, we applied the HEARRING_LOC_ICF scale retrospectively to data obtained from cohorts of normal-hearing and cochlear implant listeners at our institutes. Norm data for each setup were retrieved from normal-hearing listeners with hearing thresholds for both ears (0.250–8 kHz) within the normal range based on age and sex, as defined by the BS 6951:1988, EN 27029:1991, and ISO 7029-1984 standards. The protocol was approved by the ethics committee of the Antwerp University Hospital (15/10/96) on 15 June 2015, by the South Metropolitan Health Service Human Research Ethics Committee (RGS0000000537) on 26 September 2017, by the Ethics Committee at the Medical University of Würzburg (199/20) on 23 April 2021, and by the Institutional Review Board at the Institute of Physiology and Pathology of Hearing (IFPS:KB/02/2017r).

(a) Comparability across institutes

**Figure 2:**
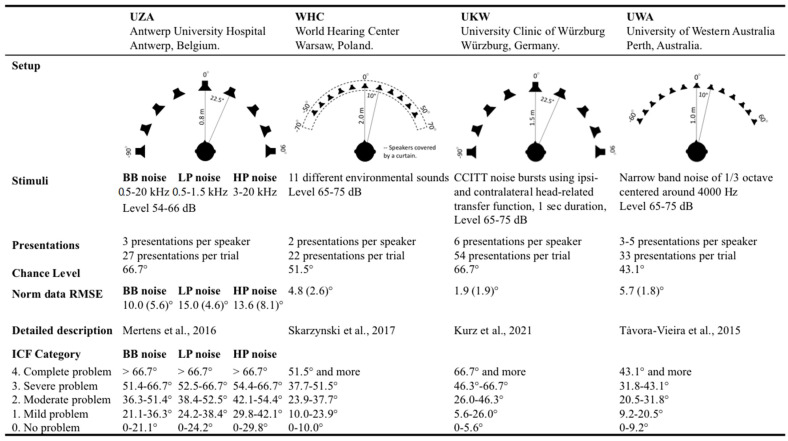
**Schematic of the variations in localization test procedures.** RMSE: root-mean-square error, BB: broadband, LP: low-pass, HP: high-pass [10,20,27,28].

To investigate comparability of localization results across institutes, SSD CI populations were chosen to illustrate the use of the HEARRING_LOC_ICF scale. The HEARRING_LOC_ICF scale was calculated for the localization testing setup of the Antwerp University Hospital (UZA) in Belgium, for the World Hearing Center (WHC) in Poland, for the University Clinic of Würzburg (UKW) in Germany, and for the University of Western Australia (UWA) in Australia. An overview of the different setups and the application of the scale per institute can be found in Figure 2 [10,20,27,28]. Data from the study from Mertens et al. [10] were used, including ten subjects with SSD, all of whom had acquired profound unilateral sensorineural hearing loss (air conduction pure tone average at 0.5, 1, and 2 kHz ≥85 dB HL) in one ear and contralateral normal hearing (air conduction pure tone average at 0.5, 1, and 2 kHz ≤ 25 dB HL). Subjects had an average of eight years’ (range 4–11) device experience at time of testing and a mean age at implantation of 48 years (range 22–62 y ±14 y). They were tested with and without the CI, with the contralateral normal-hearing ear open in both conditions. Data from the World Hearing Center included 54 SSD CI users, data from the University Clinic of Würzburg 19 SSD CI users, and data from the University of Western Australia 46 SSD CI users. All of them were tested 12 months after implantation, with and without the CI, with the contralateral normal-hearing ear open in both conditions.

(b) Comparability across localization testing setup

The procedural elements of localization testing can vary. An example of a source of procedural variation is the stimulus, which can vary in type, number, presentation level, and duration. While all other parameters remained unchanged, three different stimuli were compared in the SSD group of Mertens et al. [10]: broadband noise stimuli (BB; 0.5–20 kHz), low-pass noise stimuli (LP; 0.5–1.5 kHz), and high-pass noise stimuli (HP; 3–20 kHz). We applied the HEARRING_LOC_ICF scale on this data set to investigate the effect of stimulus type in an SSD population.

(c) Comparability across listeners

To investigate comparability of localization results across listeners using the HEARRING_LOC_ICF scale, the scale was applied on data from the SSD group and a group with Asymmetric Hearing Loss (AHL) from Mertens et al. [24]. The SSD group comprised 12 subjects with contralateral normal hearing (i.e., PTA0.5, 1, 2, and 4 kHz ≤ 30 dB HL) and the AHL group 11 subjects with contralateral mild-to-moderate hearing loss (i.e., air conduction PTA0.5, 1, 2, and 4 kHz > 30 dB HL).

## 3. Results

(a) Comparability across institutes

**Figure 3:**
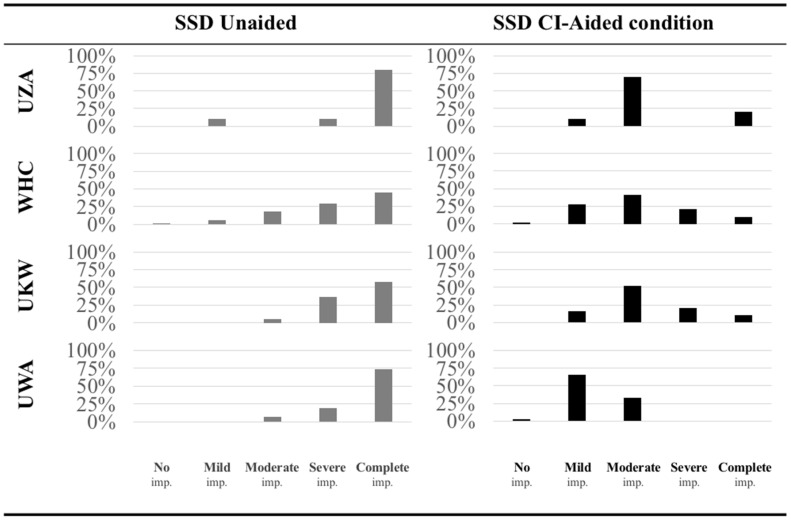
Application of the HEARRING_LOC_ICF scale on SSD CI data from the Antwerp University Hospital (UZA) in Belgium, for the World Hearing Center (WHC) in Poland, for the University Clinic of Würzburg (UKW) in Germany, and for the University of Western Australia (UWA) in Australia. Histograms are presented with the distribution of the observed localization impairments in the unaided and the CI-aided conditions.

Using the HEARRING_LOC_ICF scale, we pooled data from SSD CI recipients from different institutes (Figure 3). Overall, the majority (60%) of the SSD patients experienced *complete impairment* of sound localization in the unaided condition, measured with the HEARRING_LOC_ICF scale. In the aided condition, we observed a shift towards *moderate impairment*. The majority (42%) of all SSD CI patients experienced a *moderate impairment* with sound localization in the aided condition.

(b) Comparability across localization testing setup

**Figure 4:**
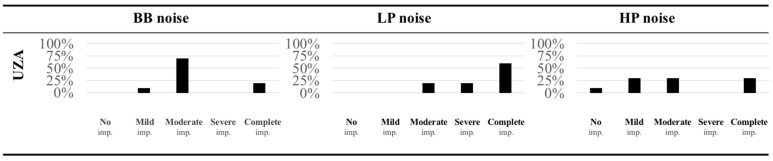
Application of the HEARRING_LOC_ICF scale on SSD CI data from the Antwerp University Hospital (UZA) for three different localization stimuli: BB noise, broadband noise; LP noise, low-pass noise; and HP noise, high-pass noise. Histograms are presented with the distribution of the observed localization impairment for the three different stimuli.

As listed in Figure 2, the mean localization error in the NH listeners is lower in the condition with the BB noise compared to the LP and the HP noise. In other words, wider-band sources are easier to localize as mentioned in the introduction. Consequently, this resulted in different HEARRING_LOC_ICF scales for the three different stimuli. After applying the HEARRING_LOC_ICF scale and thus, after correcting for the observed differences in the norm data, the SSD CI group appears to have the most difficulty with localizing LP noises. In the condition with the LP noise, the majority (60%) has *complete impairment* with sound localization. In the condition with the HP noise, the degree of observed impairment was more variable. This is in line with previous evidence that suggests that CI recipients rely mostly on ILDs and that their ITD sensitivity is generally supposed to be poorer (Noel and Eddington, 2013). In the condition with a wider-band source, a shift was observed, with the majority (70%) reporting only *moderate impairment* with sound localization (Figure 4).

(c) Comparability across listeners

**Figure 5:**
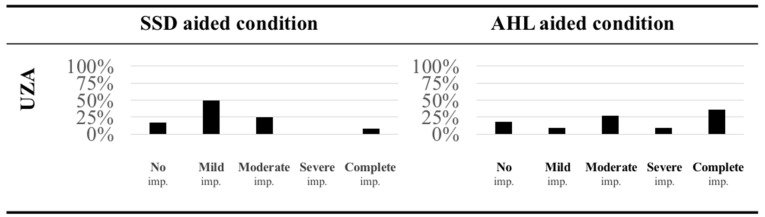
Application of the HEARRING_LOC_ICF scale on data from the Antwerp University Hospital (UZA) for two different listener groups, a single-sided deaf group (SSD) and a group with asymmetric hearing loss (AHL), retrieved from Mertens et al. [24]. Histograms are presented with the distribution of the observed localization impairment for the two different groups.

Using the HEARRING_LOC_ICF scale, we were able to demonstrate the difference in localization accuracy in the CI-aided condition between the SSD group and the patients with asymmetric hearing loss from the study from Mertens et al. [24]; see Figure 5. While the degree of localization accuracy was widely distributed in the AHL group (with the largest number experiencing *complete impairment*), the majority of the SSD group (50%) had only *mild impairment* with sound localization.

## 4. Discussion

Sound localization is an important aspect of hearing, and its preservation or restoration is a major goal of hearing implant technologies. Sound localization measurement is commonly performed both in the assessment of the rehabilitation of hearing implant users, as well as in psychophysical research to improve hearing implant performance. Across institutions, there exist major variations in localization assessment. These include variations in the testing procedure as well as in the statistical and reporting metrics used. A standardized testing procedure has been reported elsewhere [29]; however, the latter source of variation remains an issue within the audiological community. Here, the HEARRING group proposes a standardized classification system based on the ICF framework. The ICF framework is endorsed by the WHO and is used worldwide as the standard for measuring and describing health and disability (World Health Organization, 2001). Therefore, it provides a common language to define different perspectives of health (biological, psychological, and social) on an individual level, focusing on consequences of health conditions rather than causes. Using this common language, one is able to investigate the effect of an intervention (such as cochlear implantation) on different perspectives of hearing, such as audibility, speech perception, and sound localization. Danermark et al. developed the ICF core set for hearing loss in 2010, but no patient-centered CI outcome assessment protocol based on the ICF currently exists [34,35,36,37,38,39]. Therefore, we aimed to define a CI outcome assessment protocol, including all relevant ICF categories to describe the impact of cochlear implantation on different aspects of health. One of them is sound localization with code b2302. This paper introduces a standardized classification system which can categorize individuals, according to their localization performance, into one of five ICF categories based on the severity of impairment, if present. We were able to demonstrate the improvement in comparability across institutes, localization testing setups, and listeners by applying the HEARRING_LOC_ICF scale on retrospective data. For example, when we pooled data from SSD CI recipients from different institutes, the same trends across institutes were observed, with the majority of the SSD patients experiencing *complete impairment* of sound localization in the unaided condition and *moderate impairment* in the aided condition. In the majority of the SSD CI group from UWA, on the other hand, only mild impairment was observed in the aided condition. This could be explained by the intensive training program the SSD CI recipients are offered, resulting in better sound localization accuracy [40]. Earlier research in SSD patients also reported significant improvement in localization accuracy after cochlear implantation. The study of Arndt et al., for example, found an improvement from 33.9° to 15.0° in 11 SSD CI recipients [41]. Another study from Grossman et al. found an improvement from 63.2° (SD 22°) to 27.6° (SD 6.5°) in a similar SSD CI population. Since the localization testing setup was different in both studies, reported data cannot be compared or pooled. The setup from Arndt et al., for example, includes 7 speakers and uses 10 presentations per speaker (70 presentations) whereas the setup from Grossman et al. includes 9 speakers and 5 presentations per speaker (45 presentations). Both variables are known to affect the accuracy of sound localization. The standardized classification system will provide individuals such as those who are typically not familiar with the interpretation of degrees of error with a clearer understanding of the general magnitude of the localization impairment, as well as improvements derived from hearing implant use.

## 5. Conclusions

The HEARRING group hopes clinicians put this recommendation into practice. The application of our standard will help to facilitate multi-center studies, as well and allow better meta-analyses of data, resulting in improved evidence-based practice in the field.

## Data Availability

Data available on request due to restrictions eg privacy or ethical. The data presented in this study are available on request from the corresponding author.
